# Persistent oppression and simple decompression both exacerbate spinal cord ascorbate levels

**DOI:** 10.7150/ijms.41289

**Published:** 2020-05-18

**Authors:** Yawen Zhang, Guojin Hou, Wenliang Ji, Feng Rao, Rubing Zhou, Shan Gao, Lanqun Mao, Fang Zhou

**Affiliations:** 1 Department of Orthopaedics, Peking University Third Hospital, Beijing, China; 2 Beijing National Laboratory for Molecular Sciences, Institute of Chemistry, The Chinese Academy of Sciences (CAS), Beijing, China; 3 Trauma Medicine Centre, Peking University People's Hospital, Beijing, China

**Keywords:** Online electrochemical system, In vivo microdialysis, Ascorbate, Spinal cord injury

## Abstract

**Background**: Surgical decompression after acute spinal cord injury has become the consensus of orthopaedic surgeons. However, the choice of surgical decompression time window after acute spinal cord injury has been one of the most controversial topics in orthopaedics.

**Objective**: We apply an online electrochemical system (OECS) for continuously monitoring the ascorbate of the rats' spinal cord to determine the extent to which ascorbate levels were influenced by contusion or sustained compression.

**Methods**: Adult Sprague-Dawley rats (n=10) were instrumented for ascorbate concentration recording and received T11 drop spinal cord injury (SCI). The Group A (n=5) were treated with immediately decompression after SCI. The Group B (n=5) were contused and oppressed until 1 h after the injury to decompress.

**Results**: The ascorbate level of spinal cord increased immediately by contusion injury and reached to 1.62 μmol/L ± 0.61 μmol/L (217.30% ± 95.09% of the basal level) at the time point of 60 min after the injury. Compared with the Group A, the ascorbate level in Group B increased more significantly at 1 h after the injury, reaching to 3.76 μmol/L ± 1.75 μmol/L (430.25% ± 101.30% of the basal level). Meanwhile, we also found that the decompression after 1 hour of continuous compression will cause delayed peaks of ascorbate reaching to 5.71 μmol/L ± 2.69 μmol/L (627.73% ± 188.11% of the basal level).

**Conclusion**: Our study provides first-hand direct experimental evidence indicating ascorbate is directly involved in secondary spinal cord injury and exhibits the dynamic time course of microenvironment changes after continuous compression injury of the spinal cord.

## Introduction

Spinal cord injury (SCI) influences millions of people around the world and often results in permanent deficit of motor, sensory, and autonomic functions 1. The number of new SCI cases occurring in the United States is estimated to be around 11,000 each year 2. SCI is a two-stage process. Primary injury always leads to a series of cascade reactions, which is known as secondary spinal cord injury, including ischemia, inflammation and apoptosis 3. Besides, a series of acute neurochemical imbalance such as energy metabolism disorder 4, excessive oxidative stress 5 and glutamate neurotoxicity 6-8 take on an indispensable role in inducing pathological changes which caused the long-term functional and histological damage.

Compared with other human tissues, nervous tissues own the highest concentration of ascorbate 9. As one of the endogenous small-molecule antioxidants existing in the central nerve system (CNS), ascorbate is known for scavenging free radical for neuro-protection 10. It is also a coenzyme involved in a series of hydroxylation reactions regulating the synthesis of collagen and neurotransmitters 11 and have a significant role in epigenetic regulation 12. Compared with the many studies done on roles of ascorbate in the brain, concentration and effort of ascorbate in the spinal cord have been less researched. Such exploration fundamentally necessitates an analytical system that can directly interface with living system, while maintaining great selectivity and temporal/spatial resolution, as well as applicability in specific pathological processes, prerequisites for reliably capturing and further interpreting dynamics of ascorbate.

Over the past few years, surgical treatment of decompressing spinal cord has become the current mainstream treatment option for doctors. The most powerful evidence for spinal decompression experiments following spinal cord injury was proposed by Dimar et al. in 1999 13. They contused the rat thoracic spinal cord and applied continuously epidural compression. Surgical decompression was performed at 0, 2, 6, 24, and 72 hours after injury. The study found that neurological recovery was inversely related to compression time. Lee et al. also found similar results in the pig experiment 14.A large number of clinical studies have also shown that decompression therapy in patients with severe acute spinal cord injury can significantly improve neurological prognosis and involves fewer complications 15, 16. However, the question about the optimal timing of surgical interventions (or even the intervention itself) has generated considerable debate and remains unclear. Studies demonstrated that decompression reduces secondary pathological mechanisms, such as ischemia, hypotension, direct damage to the cell membrane, energy metabolism imbalance, etc. Howver detailed neurochemical mechanisms are still not fully understood.

In this paper, we, for the first time, reveal the real-time dynamic changes of ascorbate in the acute phases of spinal cord contusion injury and compression injury. By taking advantage of the electro-analytic activity of single-walled carbon nanotubes (SWNTs) toward electrochemically oxidizing ascorbate, the system has been proven to be very appropriate for analysing the changes of ascorbate independent of other co-existing interference in vivo 17.

## Methods

### Animals and study groups

**Figure 1:**
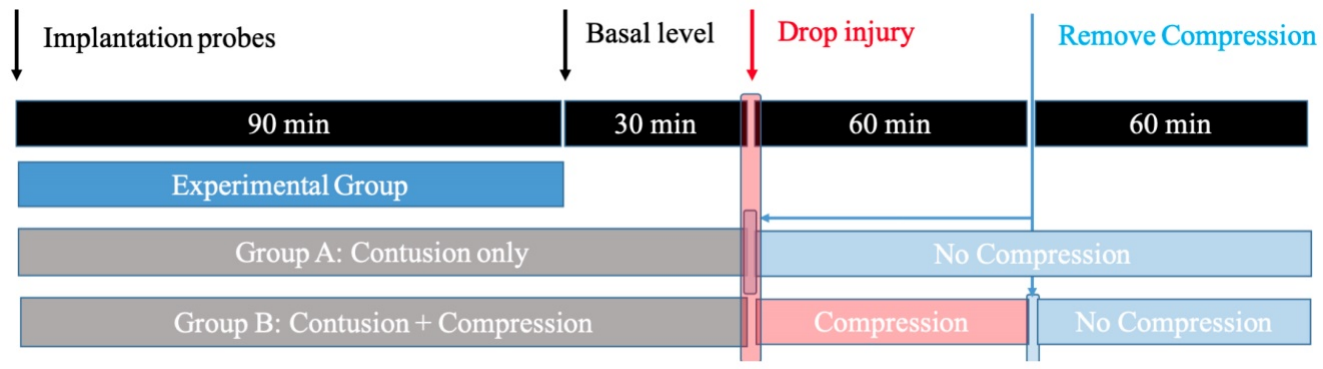
The design for the microdialysis experiment. Animals are divided into: 1) Group A, in which the impactor weighing 20 g was dropped onto the exposed cord and immediately removed; 2) Group B, in which the animals received the same weight drop injury and followed by persistent compression for 1h before decompression.

All animal protocols and procedures employed in this study were approved by the Medical Ethics Committee of Peking University Third Hospital. To study the spinal cord response induced by contusion SCI and contusion plus compression SCI, adult male Sprague-Dawley rats (weighing 250 ± 30 g at the time of surgery) were randomly divided into two experimental groups: Group A (contusion group: laminectomy + probe implantation + contusion SCI); Group B (compression group: laminectomy + probe implantation + contusion plus compression SCI). Each of group contained five rats. A schematic overview of the experimental design is illustrated in Figure 1.

### Rat model of traumatic SCI

The animals were anaesthetized with urethane (20 % w/v; 7 ml/kg ip.) and were fixed on Spinal Cord Impactor (RWD/68097, RWD Life Science Inc, China). A midline dorsal incision was performed using sterile technique. We dissected the paravertebral muscles in order to expose the T8-L2 laminae. Laminectomy was performed at T10-L1 levels. SCI was accomplished by Spinal Cord Impactor. An impactor weighing 20g, 2.50 mm in diameter, was dropped from 15 cm onto the exposed cord at T11 level. For the condition of ''contusion'', the impactor was removed from the cord immediately. For the condition of ''contusion plus compression'', following the weight drop injury, we left the impactor resting on the cord for 1h before removal, as described by Elena 18.

### Hematoxylin-eosin (HE)staining

The 5-μm tissue sections that were made from paraffin-embedded blocks of the spinal cord specimens fixed by 4% phosphate-buffered paraformaldehyde solution (4 °C), extracted from the injured animals from Group A and Group B, were subjected to routine hematoxylin and eosin staining and evaluated by microscope.

### iNOS Immunostaining

Spinal cords were embedded in paraffin, and semi serial 5-μm sections were taken horizontally. Sections were mounted on slides, rehydrated, and placed in 3% H_2_O_2_ to block endogenous peroxidase activity. The tissue was rinsed, steamed with a solution of citrate buffer for 25 min, and rinsed with 0.05 M PBS (pH 7.40). Nonspecific activity was blocked with normal horse serum. Subsequently, spinal cord sections were incubated at room temperature for 2 h in a 1:1000 dilution of rabbit polyclonal antibody against iNOS (GB11119, Servicebio Inc, China). Further rinsing was done with PBS and secondary antibody applied (GB23303, Servicebio Inc, China). After rinsing, the sections were visualized with DAB kit (G1212, Servicebio Inc, China), and the sections were then washed in distilled water and counterstained with hematoxylin.

### In Vivo Micro-dialysis and Online Electrochemical System for Ascorbate

**Figure 2:**
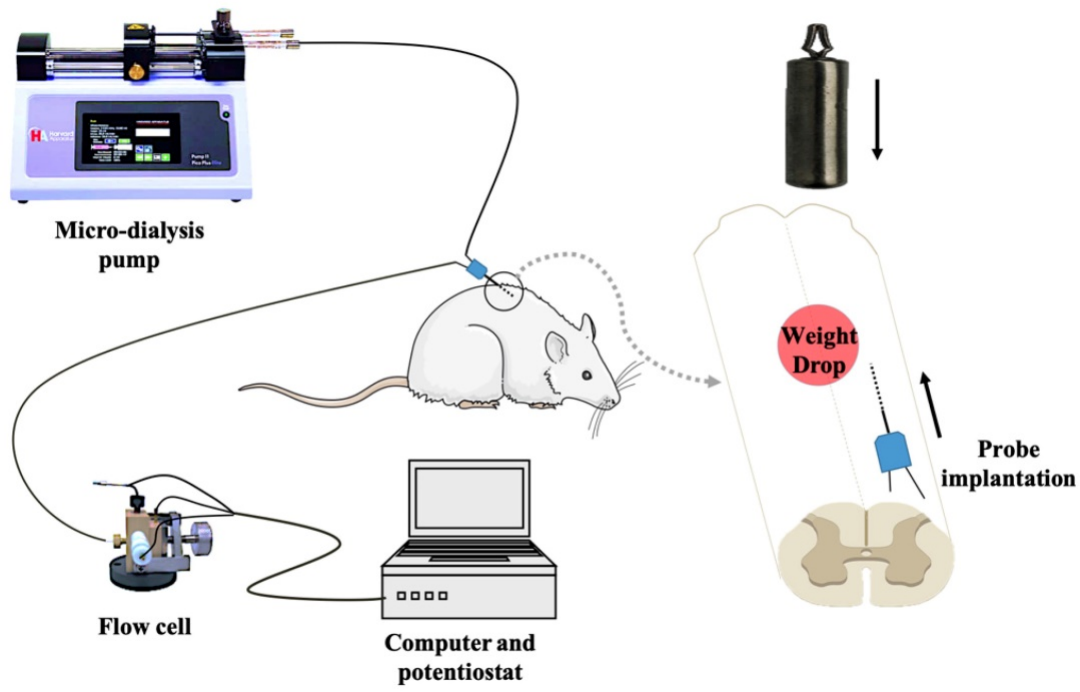
Schematic diagram of the on-line electrochemical method integrated with in vivo micro-dialysis for continuous monitoring of spinal cord ascorbate in SCI models. The micro-dialysis probe was perfused with aCSF with a flow rate of 3 μL/min. The working electrode was applied at +30 mV (vs. Ag/AgCl electrode).

In vivo micro-dialysis was performed by implanting a micro-dialysis probe (2 mm in length, CMA/12, Carnegie Medicine AB, Sweden) into the right side of spinal cord dorsal area with 30-degree horizontally from tail-to-head direction at L1 level. After equilibrating for 90 min by unceasingly perfusing with artificial cerebrospinal fluid (aCSF) at a flow rate of 3 μL/min driven by a micro injection pump, the spinal cord microdialysate was directly delivered into a thin-layer radial electrochemical flow cell for continuously detecting ascorbate. In the first 30 min, animals are independent of any intervention. We select the ascorbate average of 20 minutes before the contusion as the basal level. Then, according to the grouping conditions, continue to left the impactor on the spinal cord or remove the impactor. The on-line electrochemical analytical system consists of in vivo micro-dialysis and a thin-layer electrochemical flow cell acting as selective electrochemical detection. The thin-layer electrochemical flow cell consists of a thin layer radial flow block equipped with a glassy carbon (GC) electrode (6-mm diameter) as the working electrode, a stainless steel as counter electrode and an Ag/AgCl electrode (3 mol/L NaCl) as the reference electrode. To achieve the specificity for determining ascorbate, the GC electrode was modified with the heat-treated single-walled carbon nanotubes (SWNTs) with a method reported in our previous work^19^-^22^. The experimental setup for continuous on-line measurements of extracellular ascorbate by integrating electrochemical detection with in vivo micro-dialysis was schematically shown in Figure 2.

### Statistics

The levels of microdialysate ascorbate were reported as the percentage of their basal level. The data were reported as the mean ± standard deviation (SD). The differences of change in the concentration of ascorbate between Group A and Group B were assessed with Student's t-Test. P< 0.05 was considered as significantly different.

## Results

### Online Electrochemical System (OECS)

**Figure 3:**
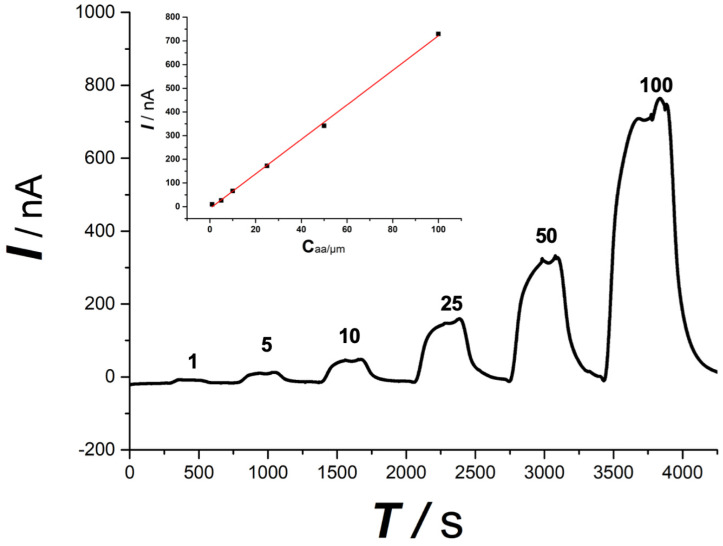
Typical linear response toward ascorbate standard solution. Ascorbate concentration was indicated in the figure. Inset, plot of current recorded vs. ascorbate concentration. The OECS system shows a good linear response toward ascorbate standard solution from 1 μM to 100 μM.

The online electrochemical method shows a linear response toward ascorbate, as shown in Figure 3, within the concentration range from 1 μmol/L to 100 μmol/L (I (nA) = 7.18 C_aa_(μmol/L)) with a linear coefficiency of 0.99. The tolerance of the OECS against the variation of O_2_ and pH during SCI was studied in our previous study in vitro by comparing the current responses for ascorbate in the micro-dialysate sampled from the aCSF with different levels of O_2_ and pH 23. The aCSF deoxygenated or acidified did not produce a significant difference in the current response of ascorbate, as compared with that in aCSF filled with ambient air and at normal physiological pH (pH 7.4). Ischemia and hypoxia, lactic acid accumulation leading to reduced pH is a classic pathological change of secondary spinal cord injury. The specificity evidence of this method applying in SCI is encouraging.

### Spinal cord Ascorbate Change after contusion or compression

**Figure 4:**
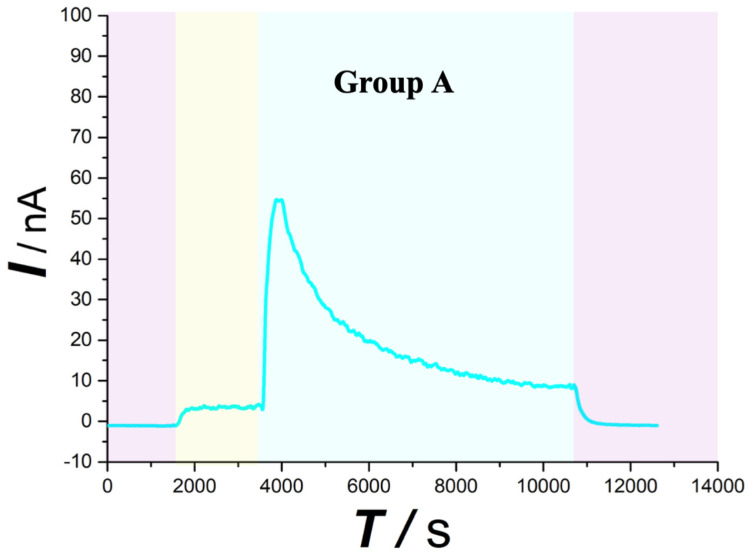
Typical current-time responses of ascorbate recorded in the microdialysates continuously sampled from spinal cord in Group A. (Purple area) Microdialysate with aCSF; (Yellow area) Microdialysate with basal level; (Blue area) Microdialysate after contusion injury. Other conditions were the same as those in Figure 3.

As typically shown in Figure 4,5 (purple area), this system was found to be very stable for continuous monitoring of aCSF. Figure 4,5 (yellow area) displays that basal ascorbate level of spinal cord without injury did not change during the first 30min after beginning the recording. After contusion SCI (Figure 4 blue area), the spinal ascorbate increases immediately and then decreases slowly.

**Figure 5:**
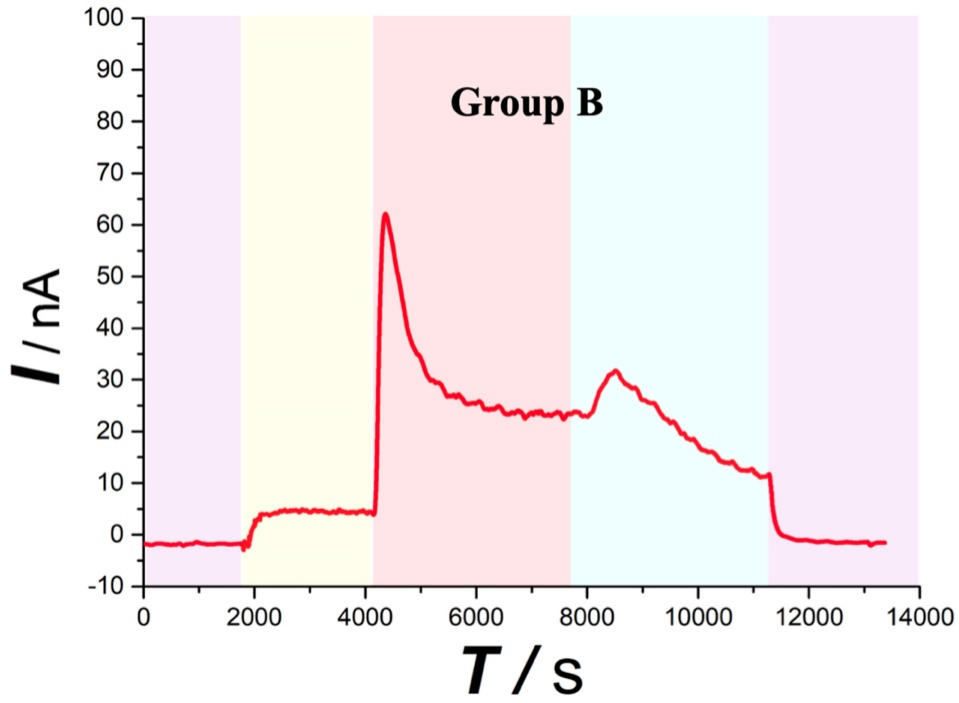
Typical current-time responses of ascorbate recorded in the microdialysates continuously sampled from the spinal cord of Group B. (Purple area) Microdialysate with aCSF; (Yellow area) Microdialysate with basal level; (Red area) Microdialysate after contusion plus compression injury; (Blue area) Microdialysate after decompression. Other conditions were the same as those in Figure 3.

Similar with the overall change trend obtained with the Group A. After contusion plus compression SCI (Figure 5 red area), the Group B spinal ascorbate increases immediately and then decreases slowly, but the speed and magnitude of the decrease are smaller than those of group A.

**Figure 6:**
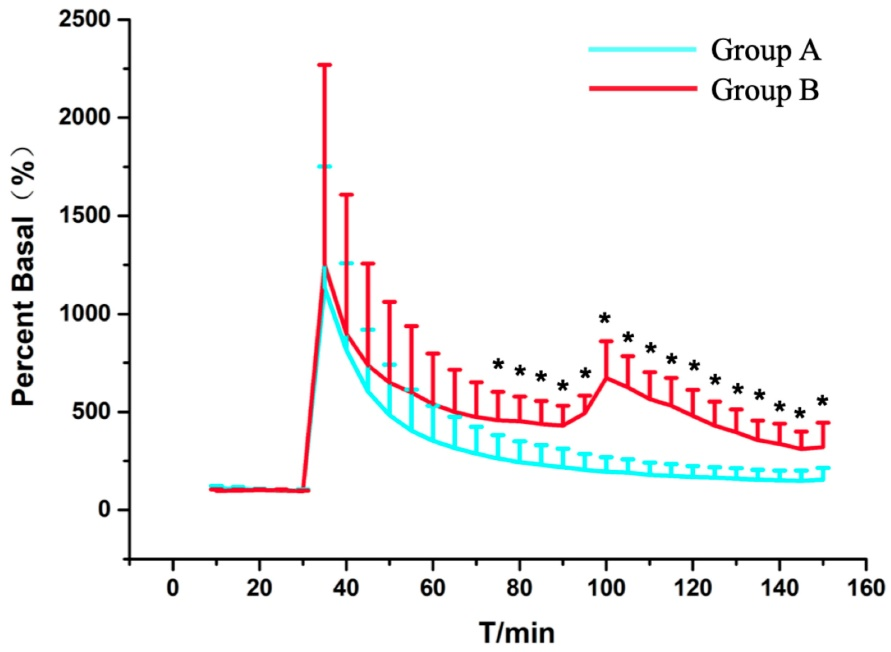
Statistical results of the changes in the levels of ascorbate in Group A (blue trace) and Group B (red trace) as a function of time. Each point represents the mean ascorbate percentage of their basal level. P < 0.05 (*) means statistical significances of differences between Group A and Group B. Other conditions were the same as those in Figure 3.

As shown in Figure 6, the spinal ascorbate of Group A finally reaches to 1.62 μmol/L ± 0.61 μmol/L (217.30% ± 95.09% of the basal level) at the time point of 60 min after SCI. With persistent compression after SCI, the ascorbate level of Group B was 3.76 μmol/L ± 1.75 μmol/L (430.25% ± 101.30% of the basal level) 1 h after SCI. To our surprise, when we decompressed the spinal cord 1 h after the injury, we found a delay peak different from the peak of the acute injury. The statistical analysis (Figure 6), also indicates the change of ascorbate levels of Group A was significant difference compared with Group B from the 45 min after SCI (P=0.04, n = 5). Although the previous studies have suggested that decompression treatment after SCI could change the concentration of small molecular substances related to energy metabolism such as glucose, lactic acid, pyruvic acid, etc. in subacute and chronic phase after SCI 18, 24, as we know, it is the first time we observed the effects of persistent compression on the SCI-induced ascorbate change in real time with our OECS.

### Persistent compression exacerbates SCI-associated damage

**Figure 7:**
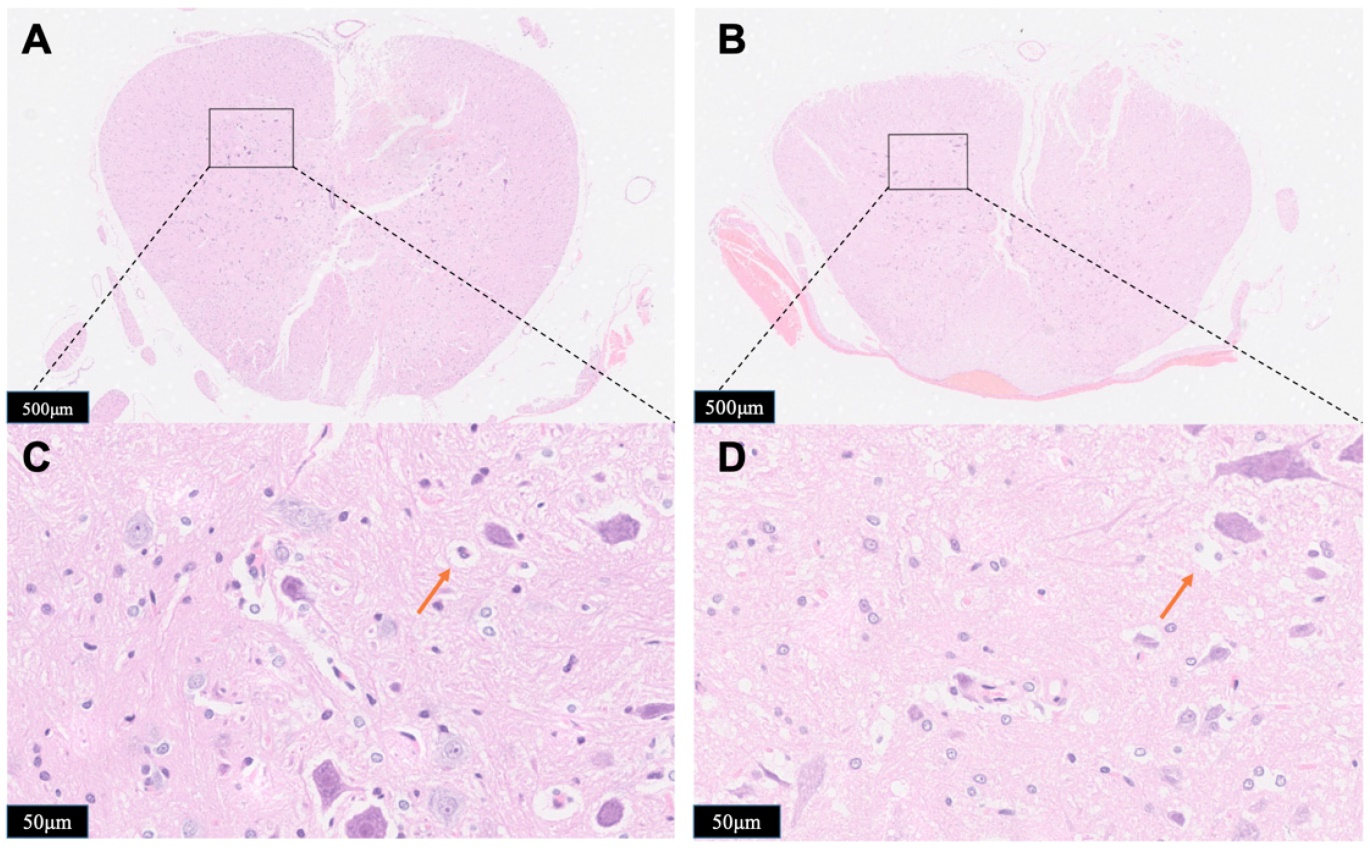
Horizontal sections taken from Group A (A, C) or Group B (B, D). The spinal cord tissue was subject to hematoxylin-eosin (HE) staining and was examined by microscope for the identification of tissue damage. Red arrows showed the vacuoles. The results presented in the text are representative.

The observation of gross histological (Figure 7A,7B) and local position (Figure 7C,7D) (the opposite side of the probe implanted portion) were given to determine the impact of compression to the spinal cord damage. Major spinal cord tissue destruction occurs at the upper impactor point and its vicinity. Significant traumatic manifestations were presented with Group B, including more hemorrhagic lesions in the gray matter, neuronal degeneration, white matter edema, more vacuoles, neurons shrink into triangles. Compared with Group B, the traumatic manifestations were obviously reduced in Group A, as validated by less vacuolation and more spared tissue.

### Immunohistology

**Figure 8:**
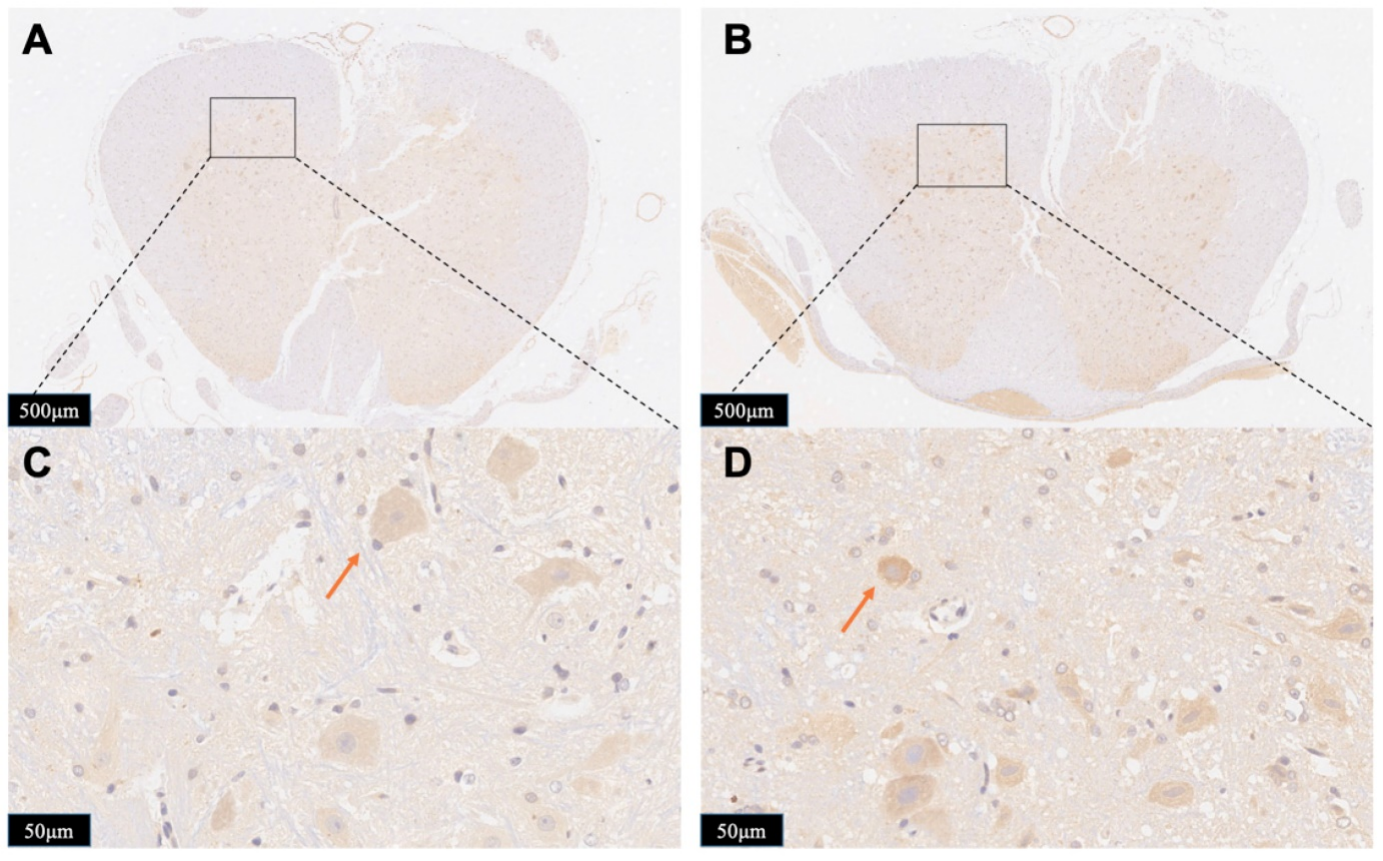
Photomicrographs showing the iNOS-positive cells in the boundary zone of the lesion area in the rats from Group A (A, C) and Group B (C, D). Staining of positive immunoreactivity was mainly localized in neurons. Red arrows showed the iNOS-positive cell. The results presented in the text are representative.

To further exhibit the neurotoxicity efficiency of persistent compression, we tested the immunoreactivity of iNOS in the spinal cord in both Group A and Group B. iNOS positive cells stained brown in cytoplasm. Figure 8 displays typical immunohistological results in spinal cord slices form Group A (Figure 8A,8C) and Group B (Figure 8B,8D). There were less detectable iNOS positive cells in the Group A slices. On contrast, after contusion plus compression injury, immunoreactivity of iNOS was obviously observed in neurons, glial cells which was indicated in the data of local position in Group B. The increased iNOS immunoreactivity in Group B demonstrates more oxidative DNA damage, more glutamate excitotoxity, and more neural damage, indicating an enhanced neurotoxicity efficiency of persistent compression.

## Discussion

This is the first report regarding the influence of simple contusion, persistent compression on extracellular ascorbate in SCI with OECS. The specificity study shown in Figure 3 demonstrates that online electrochemical method has a good selectivity and linear response for ascorbate and has been confirmed that it's very suitable for studying the changes of ascorbate independent of O_2_ and pH fluctuation and other co-existing interference in the spinal cord. As shown in our earlier research 21, 25, 26, our OECS coupled with in vivo micro-dialysis has high specificity, good accuracy, and stability for monitoring ascorbate continuously in the study of different pathophysiological mechanisms.

**Figure 9:**
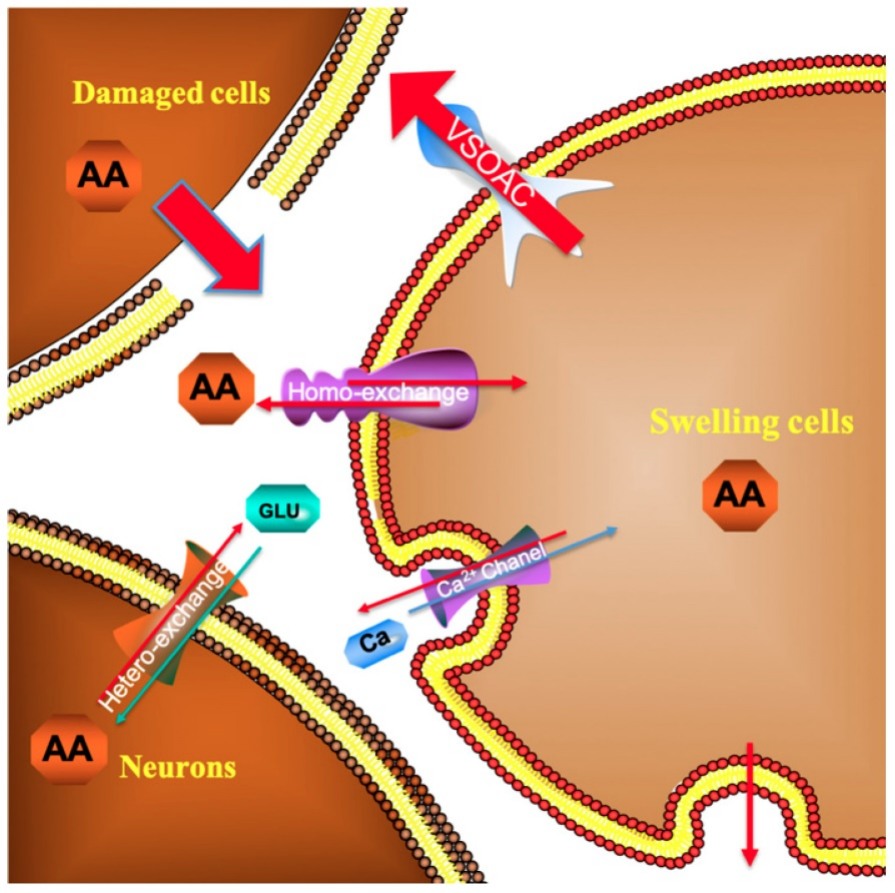
Molecular mechanism model of ascorbate involved in spinal cord secondary injury.

As depicted in Figure 4, in the first 30 min after beginning the recording, the current response recorded for the spinal cord microdialysate continuously sampled from the spinal cord remained constant indicating that after 90 minutes of equilibrating, the local microenvironment of the spinal cord reaches to steady state. In previous experiment, we have confirmed that simple probe implantation into the rat spinal cord will not cause a large change in the concentration of spinal cord ascorbic acid after equilibrating 27. This stabilization is comprehensible because the extracellular ascorbate dynamically self-regulates through homeostasis. For instance, excess ascorbate can be transported into neural cells via sodium-dependent vitamin C transporter 2 (SVCT2) 28. When the spinal cord injury occurs, extracellular ascorbate concentration in the injured area increases sharply. This extremely rapid increase is owing to the partial rupture of some cell membranes caused by the impact damage, and the intracellular high concentration of ascorbic acid instantly rushes out. Some scholars believe that after the injury, extracellular accumulation of high concentration of ascorbate is a self-protection mechanism, and the enhancement of the overall reducing ability of the extracellular fluid helps to prevent oxidative damage of the cell membrane 29. Subsequently, the ascorbate concentration slowly decreased, and the extracellular concentration increased nearly twice as much as the basal level 1 h after the injury. In response to this increase, we, for the first time, systematically give a general ascorbate molecular mechanism model related to SCI (Figure 9). The damaged cells contribute greatly to the extracellular high concentration of ascorbate after injury. The sustained transporting of the glucose transporter and sodium-dependent vitamin C transporter 2 (SVCT2) lead to a large difference in the concentration of ascorbate inside and outside the neurons and glial cells 9, 30. Volume-sensitive organic anion channels (VSOAC) are functionally defined plasma membrane anion channels relevant to regulating cell volume 31. After spinal cord injury, ion pump impediment, ion channel inactivation, ion exchange function reversal and cell depolarization cause intracellular Na+ increase, exacerbating cell edema. Volume sensitive organic anion channels (VSOACs) activated by cell edema also intensify the release of ascorbate 32, 33. In addition, glutamate transporter dysfunction, impaired glial cell reabsorption of glutamate, and sodium-dependent glutamate transporter reversal leading to the release of glutamate by neurons and glial cells. These factors together lead to an increase in extracellular glutamate concentration 34. Intracellular ascorbate efflux after injury can accelerate the reabsorption of glutamate and reduce the accumulation of extracellular glutamate for the purpose of reducing neurotoxicity. The glutamate-ascorbate hetero-exchange mechanism provides a theoretical basis for the neuroprotective effects of ascorbate, in which ascorbate is released to extracellular spaces to promote glutamate uptake into cells to alleviate excitotoxicity in some pathological processes 35, 36. After spinal cord injury, excessive activation of L-type Ca2+ channels and N-type Ca2+channels lead to Ca2+ increase in intracellular in astrocytes and oligodendrocytes, and influx of Ca2+ also enhances the out-flow of ascorbate^34^, ^37^. Some scholars have observed that extracellular ascorbate can stimulate ascorbate efflux and proposed the ascorbate-ascorbate homo-exchange mechanism 38.

As far as we know, continuous real-time observation of ascorbate changes during continuous compression injury of the spinal cord has not been reported. By leaving the impactor compression on the spinal cord for one hour, the magnitude and rate of ascorbic acid increase were more moderate than those in the simple contusion group as shown in Figure 5. After 1h continuous pression, the extracellular concentration increased to 3.76 μmol/L ± 1.75 μmol/L (430.25% ± 101.30% of the basal level). A lot of research has confirmed that a longer duration of compression injury is associated with reduced electrophysiological recovery, increased pathological changes, and significantly greater functional impairment.

However, studies on neurochemical mechanisms associated with persistent oppression have rarely been reported. The statistical data (Figure 6) revealed that after the occurrence of spinal cord injury, the ascorbic acid change trends in the two groups were the same. But the ascorbate change in the Group B was always higher than that in the Group A at the same time. This difference was statistically different from 45 minutes after injury (P=0.04, n=5). Although it represents a higher reducing power, it also reflects from the side that it suffers from greater oxidative stress damage. Pressures above the spinal cord can directly damage cell membrane and may diminish regional spinal cord blood flow, which will accelerate cell death and exacerbate cell edema. Regional hypoperfusion and ischemia can lead to dysfunction of cell mitochondria and aggravate the production of reactive oxygen species such as O2- and H2O2. Those data indicate that damage to the spinal cord depends strongly on the duration of compression. To our surprise, when we released the compression after 1 hour of continuous compression, there was a delayed peak of ascorbate in the spinal cord. Unlike the instantaneous peak of simple contusion, this peak rises more slowly and should be related to a rapid rebound and hyperemic spinal cord blood-flow response upon decompression. This also suggests that when we exert surgical decompress, we need to assist other treatments to reduce reperfusion injury. The staining data (Figures 7 and 8) confirmed that the probe was implanted into the right side of spinal cord dorsal area. This study focuses on the spinal cord injury caused by contusion and continuous compression. We chose the left spinal cord to present the details of the pathological section, which can accurately reflect the same degree of injury of different injury models of the right spinal cord, and can eliminate the bias of probe implantation. As the HE staining data shows, Group B is associated with greater histologic evidence of spinal cord edema and cell death, which also contribute to the higher extracellular ascorbate concentration. These data provide strong evidence of a sequence of secondary injury processes that are causally related to time dependent sustained cord compression. Nitric oxide (NO) is a kind of biological information transmitter and cell messenger molecule, which is mainly induced by induced nitric oxide synthase (iNOS). iNOS which not expressing under physiological conditions is usually induced by tissue stimulation. After iNOS induces activation, excessive NO is produced, which destroys tissues by oxidation and aggravates the cell damage 39. The increase of the spinal cord ascorbate caused by persistent compression reflects the more neural damage, which could be partially demonstrated by the increase of iNOS immunoreactivity. Thus, the change of spinal cord extracellular ascorbate, synchronously occurring with the change of intracellular iNOS immunoreactivity, could be potentially used as a vital biomarker to indicate the neural damage after SCI. This consistency further validates our OECS as an effective platform for in vivo evaluating pathophysiological and neurochemical mechanism of acute spinal cord injury.

## Conclusion

In summary, our study provides first-hand direct experimental evidence indicating ascorbate is directly involved in secondary spinal cord injury. Preliminary conclusions are drawn that persistent compression can greatly increase the extracellular ascorbate concentration after acute spinal cord injury. The microenvironment of the contusion and compression groups produced statistical differences in 45 minutes after the injuries. In addition, we provided a necessary experimental basis to support that simple decompression of spinal cord injury is necessary to assist other therapeutic interventions due to reperfusion injury.

## References

[1] Rao JS, Zhao C, Zhang A, Duan H, Hao P, Wei RH (2018). NT3-chitosan enables de novo regeneration and functional recovery in monkeys after spinal cord injury. Proceedings of the National Academy of Sciences of the United States of America.

[2] Jiang F, Jaja BNR, Kurpad SN, Badhiwala JH, Aarabi B, Grossman RG (2019). Acute Adverse Events After Spinal Cord Injury and Their Relationship to Long-term Neurologic and Functional Outcomes: Analysis From the North American Clinical Trials Network for Spinal Cord Injury. Critical care medicine.

[3] Tai PA, Hsu YJ, Huang WC, Chang CH, Chen YH, Huang CC (2019). Congenital exercise ability ameliorates muscle atrophy but not spinal cord recovery in spinal cord injury mouse model. Int J Med Sci.

[4] Braughler JM, Hall ED (1984). Effects of multi-dose methylprednisolone sodium succinate administration on injured cat spinal cord neurofilament degradation and energy metabolism. Journal of neurosurgery.

[5] Khan M, Sakakima H, Dhammu TS, Shunmugavel A, Im YB, Gilg AG (2011). S-nitrosoglutathione reduces oxidative injury and promotes mechanisms of neurorepair following traumatic brain injury in rats. Journal of neuroinflammation.

[6] Huang Z, Filipovic Z, Mp N, Ung C, Troy EL, Colburn RW (2017). AC105 Increases Extracellular Magnesium Delivery and Reduces Excitotoxic Glutamate Exposure within Injured Spinal Cords in Rats. Journal of neurotrauma.

[7] Umebayashi D, Natsume A, Takeuchi H, Hara M, Nishimura Y, Fukuyama R (2014). Blockade of gap junction hemichannel protects secondary spinal cord injury from activated microglia-mediated glutamate exitoneurotoxicity. Journal of neurotrauma.

[8] Regan RF, Choi DW (1991). Glutamate neurotoxicity in spinal cord cell culture. Neuroscience.

[9] Harrison FE, May JM (2009). Vitamin C function in the brain: vital role of the ascorbate transporter SVCT2. Free radical biology & medicine.

[10] Xiao T, Wang Y, Wei H, Yu P, Jiang Y, Mao L (2019). Electrochemical Monitoring of Propagative Fluctuation of Ascorbate in the Live Rat Brain during Spreading Depolarization. Angew Chem Int Ed Engl.

[11] May JM, Qu ZC, Nazarewicz R, Dikalov S (2013). Ascorbic acid efficiently enhances neuronal synthesis of norepinephrine from dopamine. Brain Res Bull.

[12] Xue JH, Chen GD, Hao F, Chen H, Fang Z, Chen FF (2019). A vitamin-C-derived DNA modification catalysed by an algal TET homologue. Nature.

[13] Dimar JR 2nd, Glassman SD, Raque GH, Zhang YP, Shields CB (1999). The influence of spinal canal narrowing and timing of decompression on neurologic recovery after spinal cord contusion in a rat model. Spine.

[14] Lee JH, Jones CF, Okon EB, Anderson L, Tigchelaar S, Kooner P (2013). A novel porcine model of traumatic thoracic spinal cord injury. Journal of neurotrauma.

[15] Campagnolo DI, Esquieres RE, Kopacz KJ (1997). Effect of timing of stabilization on length of stay and medical complications following spinal cord injury. The journal of spinal cord medicine.

[16] McLain RF, Benson DR (1999). Urgent surgical stabilization of spinal fractures in polytrauma patients. Spine.

[17] Zhang M, Liu K, Gong K, Su L, Chen Y, Mao L (2005). Continuous on-line monitoring of extracellular ascorbate depletion in the rat striatum induced by global ischemia with carbon nanotube-modified glassy carbon electrode integrated into a thin-layer radial flow cell. Analytical chemistry.

[18] Okon EB, Streijger F, Lee JH, Anderson LM, Russell AK, Kwon BK (2013). Intraparenchymal microdialysis after acute spinal cord injury reveals differential metabolic responses to contusive versus compressive mechanisms of injury. Journal of neurotrauma.

[19] Li L, Zhang Y, Hao J, Liu J, Yu P, Ma F (2016). Online electrochemical system as an in vivo method to study dynamic changes of ascorbate in rat brain during 3-methylindole-induced olfactory dysfunction. The Analyst.

[20] Lin Y, Yu P, Hao J, Wang Y, Ohsaka T, Mao L (2014). Continuous and simultaneous electrochemical measurements of glucose, lactate, and ascorbate in rat brain following brain ischemia. Analytical chemistry.

[21] Liu K, Yu P, Lin Y, Wang Y, Ohsaka T, Mao L (2013). Online electrochemical monitoring of dynamic change of hippocampal ascorbate: toward a platform for in vivo evaluation of antioxidant neuroprotective efficiency against cerebral ischemia injury. Analytical chemistry.

[22] Gao X, Yu P, Wang Y, Ohsaka T, Ye J, Mao L (2013). Microfluidic chip-based online electrochemical detecting system for continuous and simultaneous monitoring of ascorbate and Mg2+ in rat brain. Analytical chemistry.

[23] Liu K, Lin Y, Xiang L, Yu P, Su L, Mao L (2008). Comparative study of change in extracellular ascorbic acid in different brain ischemia/reperfusion models with in vivo microdialysis combined with on-line electrochemical detection. Neurochemistry international.

[24] Zhang Y, Hillered L, Olsson Y, Holtz A (1993). Time course of energy perturbation after compression trauma to the spinal cord: an experimental study in the rat using microdialysis. Surgical neurology.

[25] Xiong S, Song Y, Liu J, Du Y, Ding Y, Wei H (2019). Neuroprotective effects of MK-801 on auditory cortex in salicylate-induced tinnitus: Involvement of neural activity, glutamate and ascorbate. Hear Res.

[26] Du Y, Liu J, Jiang Q, Duan Q, Mao L, Ma F (2017). Paraflocculus plays a role in salicylate-induced tinnitus. Hear Res.

[27] Zhang Y, Lv Y, Ji W, Zhou R, Gao S, Zhou F (2019). Therapeutic hypothermia effectively reduces elevated extracellular ascorbate concentrations caused by acute spinal cord injury. Artificial cells, nanomedicine, and biotechnology.

[28] Meredith ME, Harrison FE, May JM (2011). Differential regulation of the ascorbic acid transporter SVCT2 during development and in response to ascorbic acid depletion. Biochemical and biophysical research communications.

[29] Moor E, Kohen R, Reiter RJ, Shohami E (2001). Closed head injury increases extracellular levels of antioxidants in rat hippocampus in vivo: an adaptive mechanism?. Neuroscience letters.

[30] Rice ME, Perez-Pinzon MA, Lee EJ (1994). Ascorbic acid, but not glutathione, is taken up by brain slices and preserves cell morphology. Journal of neurophysiology.

[31] Siushansian R, Tao L, Dixon SJ, Wilson JX (1997). Cerebral astrocytes transport ascorbic acid and dehydroascorbic acid through distinct mechanisms regulated by cyclic AMP. Journal of neurochemistry.

[32] Lane DJ, Lawen A (2013). The glutamate aspartate transporter (GLAST) mediates L-glutamate-stimulated ascorbate-release via swelling-activated anion channels in cultured neonatal rodent astrocytes. Cell Biochem Biophys.

[33] Wilson JX, Peters CE, Sitar SM, Daoust P, Gelb AW (2000). Glutamate stimulates ascorbate transport by astrocytes. Brain research.

[34] Li S, Mealing GA, Morley P, Stys PK (1999). Novel injury mechanism in anoxia and trauma of spinal cord white matter: glutamate release via reverse Na+-dependent glutamate transport. The Journal of neuroscience: the official journal of the Society for Neuroscience.

[35] Rice ME (2000). Ascorbate regulation and its neuroprotective role in the brain. Trends Neurosci.

[36] Dutta A, Gautam R, Chatterjee S, Ariese F, Sikdar SK, Umapathy S (2015). Ascorbate protects neurons against oxidative stress: a Raman microspectroscopic study. ACS Chem Neurosci.

[37] Miele M, Boutelle MG, Fillenz M (1994). The physiologically induced release of ascorbate in rat brain is dependent on impulse traffic, calcium influx and glutamate uptake. Neuroscience.

[38] May JM, Qu ZC (2009). Ascorbic acid efflux and re-uptake in endothelial cells: maintenance of intracellular ascorbate. Molecular and cellular biochemistry.

[39] Lemarchant S, Dunghana H, Pomeshchik Y, Leinonen H, Kolosowska N, Korhonen P (2016). Anti-inflammatory effects of ADAMTS-4 in a mouse model of ischemic stroke. Glia.

